# S11-4: Reaching people with a disability in a small town–a practice perspective

**DOI:** 10.1093/eurpub/ckae114.249

**Published:** 2024-09-26

**Authors:** Seamus Nugent

**Affiliations:** Sports Inclusion Development Officer, Kilkenny Recreation and Sports Partnership, Ireland

## Abstract

**Purpose:**

It is well documented that people living with a disability often have restricted opportunities to participate in physical activity and may face significant barriers. This presentation provides a practice perspective on reaching, and providing inclusive, meaningful physical activity opportunities for children and adults living with disability in Ireland.

**Project or policy description:**

While significant efforts have been made in Ireland and elsewhere to facilitate access, this presentation will provide insights from a Sports Inclusion Development Officer, working in a town in Ireland, on how providing facilitators (funding streams and a varied programme of activities) does not necessarily remove barriers for people with disability to access physical activity and participate.

**Conclusions:**

There are problems in areas with low population density – small numbers of people with specific disabilities leading to no specific activities for specific disabilities. Clubs are providing options, in some instances, but they are often not easy to implement as the volunteer base has declined and many sports clubs focus on performance rather than participation. Sometimes they are difficult for those with disabilities to access and participate. Often, the policy and willingness for change is there at organisational level but at intervention level there are difficulties in implementation.

